# Supplementary material for: Nerve conduction, latency, and its association with hand function in young men
Source: PLoS One. 2024 Sep 30;19(9):e0310813. doi: 10.1371/journal.pone.0310813 (PMC11441660; doi:10.1371/journal.pone.0310813)
Supplement: Attachment — Submitted filename: Response to reviewer(s)0827.docx [file pone.0310813.s003.docx]

Dear Solna Carreon Santos from PLOS ONE

Greetings,

Kindly find my responses to your comments.

***“1. Your ethics statement should only appear in the Methods section of your manuscript. If your ethics statement is written in any section besides the Methods, please delete it from any other section.”***

The statement was deleted since it is written in the methods section.

***“2. In the online submission form, you indicated that [The data used to generate the findings of this study are available from the corresponding author upon reasonable request.].***

***All PLOS journals now require all data underlying the findings described in their manuscript to be freely available to other researchers, either 1. In a public repository, 2. Within the manuscript itself, or 3. Uploaded as supplementary information.***

***This policy applies to all data except where public deposition would breach compliance with the protocol approved by your research ethics board. If your data cannot be made publicly available for ethical or legal reasons (e.g., public availability would compromise patient privacy), please explain your reasons on resubmission and your exemption request will be escalated for approval.”***

All data underlying the findings described in the manuscript are available and uploaded as supplementary information.

***“3. Please upload a copy of Figures 1, 2 and 3 which you refer to in your text on pages 8 an 12. Or if the figure is no longer to be included as part of the submission please remove all reference to it within the text.”***

Figure 1, 2 and 3 were uploaded in the text in the right pages.

Kindest Regards

Awad M. Almuklass, PhD
